# The middle-aged and older Chinese adults’ health using actigraphy in Taiwan (MOCHA-T): protocol for a multidimensional dataset of health and lifestyle

**DOI:** 10.1186/s12889-023-17552-0

**Published:** 2024-01-04

**Authors:** Ching-Ju Chiu, Szu-Yu Hou, Chih-Liang Wang, Hsiao-Han Tang, Po-Ching Kuo, Sheng-Fu Liang, Pei-Fen Kuo

**Affiliations:** 1 Institute of Gerontology, College of Medicine, National Cheng Kung University, Tainan, Taiwan; 2 Department of Computer Science and Information Engineering, National Cheng Kung University, Tainan, Taiwan; 3 Department of Geomatics, National Cheng Kung University, Tainan, Taiwan

**Keywords:** Aging, Lifestyle, Taiwan, Population health, Research design

## Abstract

**Background and objectives:**

Older adults keep transforming with Baby Boomers and Gen Xers being the leading older population. Their lifestyle, however, is not well understood. The middle-aged and older Chinese adults’ health using actigraphy in Taiwan (MOCHA-T) collected both objective and subjective data to depict the health and lifestyle of this population. The objectives, design, and measures of the MOCHA-T study are introduced, and the caveats and future directions related to the use of the data are presented.

**Methods:**

People aged 50 and over were recruited from the community, with a subset of women aged 45–49 invited to supplement data on menopause and aging. Four instruments (i.e., self-reported questionnaires, diary, wrist actigraphy recorder, and GPS) were used to collect measures of sociodemographic, health, psychosocial, behavioral, temporal, and spatial data.

**Results:**

A total of 242 participants who returned the informed consent and questionnaires were recruited in the MOCHA-T study. Among them, 94.6%, 95.0%, and 25.2% also completed the diary, actigraphy, and GPS data, respectively. There was almost no difference in sociodemographic characteristics between those with and without a completed diary, actigraphy, and GPS data, except for age group and educational level for those who returned completed actigraphy data.

**Conclusion:**

The MOCHA-T study is a multidimensional dataset that allows researchers to describe the health, behaviors, and lifestyle patterns, and their interactions with the environment of the newer generation of middle-aged and older adults in Taiwan. It can be compared with other countries with actigraphy and GPS-based lifestyle data of middle-aged and older adults in the future.

## Background

The aging population is growing faster around the world. At the same time, the health [1, 2], lifestyle, behaviors [3, 4], and attitudes towards later life of middle-aged and older adults [5] are changing. Numerous studies have found that factors associated with the health and quality of life of middle-aged and older adults from a variety of perspectives, such as chronic diseases, medications, psychosocial correlates, activity and engagement, physical activity, dietary, subjective age, and neighborhood environment [6–12]. To establish a comprehensive understanding that connects individual, social, and environmental determinants of health and lifestyle from diverse viewpoints, it is imperative to develop a dataset that integrates a variety of data sources.

Multidimensional investigation has gained increasing acceptance as a way of collecting personal behavioral and lifestyle data from a variety of sources that can fill the gap in assessing the health promotion needs of the aging population [13, 14]. Subjective data from middle-aged and older adults captures their feelings, beliefs, and daily behaviors. Concurrently, objective data, including actigraphy and global positioning system (GPS) technologies, measure and signify their repetitive behavior patterns [15–17]. Actigraphy provides 24-hour measurements of sleep, physical activity, and circadian rhythm [17, 18], while GPS presents passive and precise collection on mobility [15]. The use of different instruments and methods to gather both subjective and objective data broadens the possibility of identifying the association between lifestyles and health status. This approach provides a better explanation of the complexity of real life [19–21].

The majority of health studies with multidimensional data have been conducted in European and Northern American countries. For example, the Rotterdam Study in the Netherlands included objective measurements of activity and sleep using wrist actigraphy recordings in a follow-up survey with a group of 2632 adults aged 45 and above since 2004 and analyzed the physical and mental health [22]. Another example of a cross-national cohort study, Contrasted Urban settings for Healthy Aging (CURHA), used a variety of research tools, such as a map-based questionnaire, GPS, and actigraphy to capture different aspects of the lives of 175 middle-aged and older adults in Canada, 634 in France, and 500 in Luxembourg, and to explore the interaction between people and environment [23]. The above study demonstrated the value and potential of adding diverse and objective data to health research, which can improve the accuracy of identifying health and lifestyle patterns.

Chinese culture, however, is different from Western culture, and the health and lifestyle of Chinese older adults are not as well understood as in Western countries. A holistic view of health, encompassing physical, psychological, and spiritual aspects, forms the basis of Chinese health beliefs [24]. The adoption of a natural, harmonious, and balanced way based on Taoism is ingrained in Chinese adults, shaping their understanding of health and influencing their health-related decisions and actions [24, 25]. Furthermore, familism, a core value of Confucianism, strongly influences medical decision-making, health behaviors, health care, and mental health [26]. These cultural factors may lead to different lifestyles and behaviors for older adults. Specifically, there have been found that self-reported health [27], health beliefs [28], leisure activities [29], healthy lifestyles, and unhealthy behaviors [30] differ between Chinese and Westerners. Therefore, there is a need to create a dataset focused on the Chinese population to identify and compare the impact of the Chinese social and cultural context on health and lifestyle.

Furthermore, previous studies of aging have mostly focused on those in later adulthood, the Silent Generation, and beyond. While academics and policymakers have emphasized health management and life planning for the aging Baby Boomers, little attention has been paid to Generation X, who are approaching old age. The Baby Boomers were born between 1946 and 1964. Due to post-war political, economic, and sociocultural changes, they have more opportunities for formal education and employment, and their needs are more diverse than their predecessors [31, 32]. Generation X, born between 1965 and 1980s, experienced the women’s rights movement, the energy crisis, and the first introduction of personal computers, and had a better life and more choices of higher education and jobs than Baby Boomers [33]. Because of their different developmental backgrounds, Baby Boomers and Gen Xers have different life values [34], health behaviors [35, 36], social participation [37], use of healthcare services [38], consumer behaviors [39], and the ability to use technology [40] than previous generations. The accumulation of these values and behaviors has some impact on health and lifestyles in their later life. There have been found that self-reported health status [35], obesity [41], and health determinants [42] differ among the Silent Generation, Baby Boomers, and Generation X. In contrast, research on Baby Boomers and Gen Xers in Taiwan is relatively limited, focusing almost exclusively on retirement [43] and experiences, preferences, and needs for specific services and products [44–46]. There is a gap in examining the generational differences in health, lifestyles, and their correlates between the Silent Generation, Baby Boomers, and Generation X of Chinese adults in Taiwan.

Research on the application of actigraphy to the health and lifestyle of middle-aged and older Chinese adults is still in its infancy in Taiwan. There is also a lack of understanding regarding the relationship between the environment and health outcomes in the aging population. The limited data from the current population health datasets in Taiwan have not been able to reflect or identify the diversity of health status and lifestyles presented due to individual differences. This inadequacy contributed to the difficulties in developing technologies for health promotion and living assistance services for older adults. Accordingly, the middle-aged and older Chinese adults’ health using actigraphy in Taiwan (MOCHA-T), is being conducted to investigate the health and lifestyle of adults aged 50 and over using multidimensional data collection. The present study described the objectives, design, and measures of the MOCHA-T study, and also provided caveats and future directions related to the data use.

## Methods

### Study design

The MOCHA-T study is an observational study initiated in 2021 with multiple methods of data collection aiming to measure both subjective and objective data on physical, mental, and social health, as well as the behaviors and lifestyles of middle-aged and older Chinese adults in Taiwan.

### Population and samples

Individuals aged 50 years and over who met the following inclusion criteria were eligible for the MOCHA-T study: (1) able to communicate in Chinese; (2) living in the communities; (3) completing informed consent; (4) owning a smartphone. To investigate menopause and women’s health, a subset of women aged 45–49 years old were also recruited. The exclusion criteria were living in the caring institutions, being bedridden, and being unable to complete the survey due to cognitive impairment.

Participants were selected by the following steps: First, one to two counties/cities in northern, central, and southern Taiwan were selected as primary sampling units. Second, cluster and stratified sampling without sample lists were used to select the communities in each county/city. Lastly, participants were invited in each community with convenience sampling. One-paged recruitment posters were posted on the LINE accounts of different formal and informal community and social groups, which contained a brief introduction of the MOCHA-T study, the inclusion and exclusion criteria, and the quick response (QR) code of contact information. People who responded to the invitation would be arranged for an interview with a trained interviewer. Furthermore, participants were also recruited from a gynecology clinic to gather more information from women experiencing menopause. The sample size estimated for this study was based on previous study protocol using multiple study tools, including actigraphy and GPS [23]. The estimated sample size is in the range of 150 to 250 middle-aged and older adults, and the final sample size was 242.

### Pilot study

The pilot study aimed to test the study protocol, data collection instruments, and data measurements. It was conducted informally by trained interviewers. All interviewers in the pilot study were researchers in this study and received standard interviewer training from the supervisor.

Four women and two men aged 50 years and above were invited to join the pilot study in March 2021. Based on feedback received from the participants, certain adjustments to the study design and measurements were implemented. For example, modifying the format of the questionnaire and diary to make it easier for subjects to complete, revising the words of the question to improve the clarity of questions, and adjusting the wrist actigraphy recorder straps for comfort when wearing it.

### Data collection

The formal data collection was carried out between April 2021 and December 2022. The participants were interviewed face-to-face by trained interviewers. The interviewers provided detailed information on the MOCHA-T study to each participant, including the objectives, parameters, experimental duration, the rights and welfare of participants, and tool management. Participants who voluntarily consented to collaborate with the study were instructed to complete the following tasks.

On the first day of the experiment, participants were requested to fill out a structured questionnaire and were instructed on how to wear a wrist actigraphy recorder, use their smartphone to set up a GPS tracker and fill out a 7-day diary. For the next seven consecutive days, participants were required to wear an actigraphy recorder, carry their smartphone, and keep a diary. On the third day, the interviewers contacted the participants via LINE to learn about their usage. On the seventh day, interviewers retrieved participants’ actigraphy recorders and diaries and exported their GPS data from their smartphones. Participants who were not available for face-to-face interviews due to their place of residence would receive a kit by mail, including a questionnaire, a 7-day diary, a wrist actigraphy recorder, and instructions on detailed procedures. To mitigate potential differences in outcomes due to different intervention methods, the interviewers explained the study to participants through audio or video calls after they received the kit. In addition, the participants who received the kit were also able to contact the interviewers via LINE in case they had any questions during the assessment.

The MOCHA-T study was approved by the Institutional Review Board National Cheng Kung University Hospital, Taiwan (IRB No: B-ER-109-362). All procedures were conducted according to the Declaration of Helsinki.

**Fig. 1:**
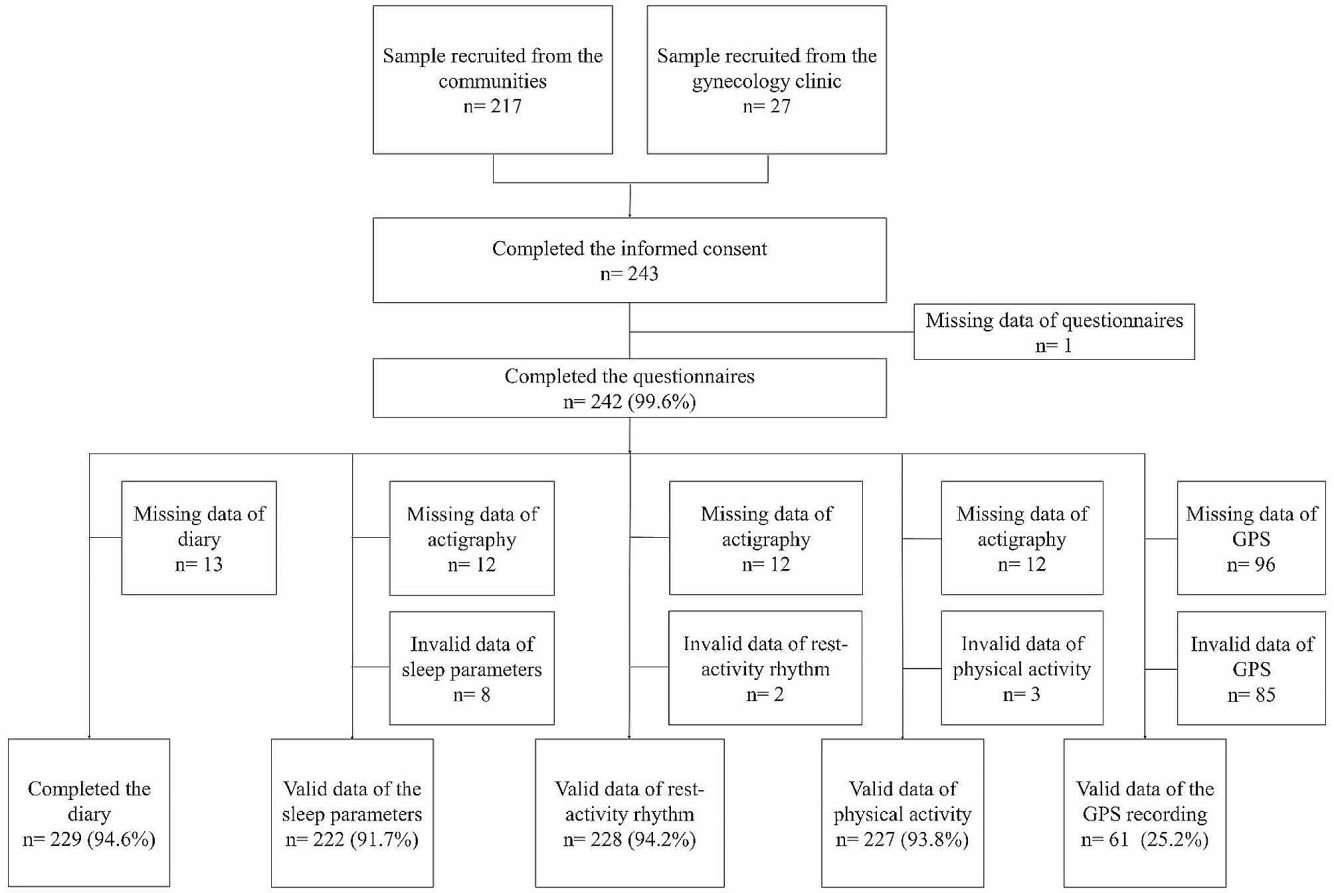
The detailed sample size of the MOCHA-T study

Figure 1 displays the final sample size from each measuring method. More than 90% of participants completed the diary and actigraphy measures. However, there were only 61 participants completed the GPS data collection (completion rate: 25.2%). Of the 181 participants without complete GPS data, 96 refused to have their GPS data recorded and 85 agreed to the recording but the data did not meet the valid criteria.

### Data measurements

Data were gathered from multiple sources, including subjective measurements (self-reported questionnaires and diary) and objective measurements (wrist actigraphy recorders and GPS recorders). Because of the different content of the questionnaire, men and women will receive different versions of the questionnaire. Table 1 shows the entire information of data measurements in the MOCHA-T study.

**Table 1:** Measurements and variables collected in the MOCHA study

Method	Measure of interest	Variables
Subjective measurements		
Self-reported questionnaire	Sociodemographic characteristics	Birth year, educational level, marital status, living arrangements, employment status, occupation, disposable income
	Health condition	Self-reported health status, chronic diseases, medications, supplements, subjective mental age, subjective physical age, height
	Exercise	Exercise frequency, exercise intensity, exercise location, types of exercise
	Social participation	Participation frequency of social clubs, religious groups, voluntary groups, learning groups, and entertainment groups
	Dietary and nutrition	Diet habits, patterns of daily food consumption, meal timing, frequency of having late-night snacks, frequency of eating out, frequency of having home-cooked foods, frequency of low-carb diets, frequency of high-protein diets, frequency of high-fiber diets, frequency of having soy foods, types of special diets
	Gynecological health (only women)	Menopausal status, history of hormone replacement therapy, history of gynecological diseases and surgery, self-reported symptoms
	Medication beliefs	Overuse belief, harm belief
	Mental health	Loneliness, depressive symptoms, life satisfaction
Diary	Sleep log	Wake-up time, bedtime, times of wake after sleep onset, sleep quality, stimulants affecting sleep intake, sedative medications
	Daily activities	Activities, time of activity
	Daily mood	Daily mood
Objective measurements		
	Anthropometry	Weight, waist circumference
Actigraphy	Sleep parameters	Sleep onset time, Wake after sleep onset, total sleep time, total wake time, sleep efficiency
	Rest-activity rhythms	MESOR, amplitude, acrophase, interdaily stability, intradaily variability, L5, L5 midpoint, M10, M10 midpoint, relative amplitude, chronotype
	Physical activities	MVPA time, LPA time, SED time, mean of physical activity counts in the morning, mean of physical activity counts in the afternoon, mean of physical activity counts in the evening, mean of physical activity counts in a day
GPS	POI categories	Hospital, clinic, café, bank, accommodation, school, supermarket, shopping mall, attractivity, cinema, activity center, park
	POI stay time	Hospital staying time, clinic staying time, café staying time, bank staying time, accommodation staying time, school staying time, supermarket staying time, shopping mall staying time, attractivity staying time, cinema staying time, activity center staying time, park staying time
	Activity space	Daily path area, standard deviational ellipse area, average distance from home

*Note*: MESOR: midline estimating statistic of rhythm; L5: least active 5-h period; M10: most active 10-h period; MVPA: moderate to vigorous physical activity; LPA: light physical activity; SED: sedentary behavior; POI: points of interest

1. Subjective measurements

Sociodemographic characteristics: The 7 questions of this sub-questionnaire included birth year, educational level, marital status, living arrangements, employment status, occupation, and disposable income.

Health condition: This sub-questionnaire comprised 7 questions to measure various aspects of health, including (1) self-reported health status: participants rated their health status on a 4-point scale, ranging from poor to very well, providing an assessment of their overall health, (2) chronic diseases: participants reported the presence or absence of physician-confirmed chronic diseases, including hypertension, hyperlipidemia, diabetes, heart diseases, cancer, depression, dementia, arthritis, osteoporosis, and others, (3) medications: participants were asked to indicate whether they were currently taking medications for the aforementioned chronic diseases, and report the total number of medications, (4) supplements: participants reported whether they take medications or nutritional supplements not prescribed by a physician, (5) subjective mental age, which assessed the difference between the participant’s perception of their age based on their mental state and their chronological age. A 9-point visual analog scale was used, ranging from − 30 (indicating they feel their mental age is 30 years younger than their chronological age), -20, -10, -5, 0, + 5, +10, + 20, to + 30 (indicating they feel their mental age is 30 years older than their chronological age), (6) subjective physical age, which assessed the difference between the participant’s perception of their age based on their physical condition and function and their chronological age using the same 9-point visual analog scale mentioned above, and (7) height, which is assessed by participants providing their height information.

Exercise: The sub-questionnaire asked about frequency of exercise in the past week (0 (never) to 4 (every day)), intensity of exercise (neither breathless nor sweating/ breathless but no sweating/ no breathless but sweating/ breathless and sweating), and preference for exercise location (indoor/ outdoor/ both). There is also an open-ended question about their usual types of exercise.

Social participation: It was evaluated using a structured questionnaire used in a previous study, which was based on the Survey of Health and Living Status of the Elderly in Taiwan [47]. In this questionnaire, participants answered the question “How often do you currently join the meetings in one of these groups or participated in such activities in a week?” for five types of groups and activities, including social club, religious group, voluntary group, learning group, and entertainment group [47]. There was a 5-point scale for each item, ranging from 1 (0 times per week) to 5 (7 times per week).

Dietary and nutrition: This 11-item sub-questionnaire assessed participants’ diet habits, patterns of daily food consumption, and meal timing. Regarding to dietary behaviors, participants were asked about the frequency of having late-night snacks, eating out, and having home-cooked foods. Furthermore, the consumption frequency of 4 types of dietary foods, including low-carb diets, high-protein diets, high-fiber diets, and soy foods was also assessed. Lastly, participants were asked whether they are conducting special diets, including intermittent fasting, dietary approaches to stop hypertension (DASH diet), gluten-free diets, ketogenic diets, Mediterranean diets, and others. This sub-questionnaire was validated by experts in the field of nutrition and geriatric care.

Gynecological health (only women): Female participants were asked about their current menopausal status, history of hormone replacement therapy, history of gynecological diseases, and history of gynecological surgery. In addition, the Menopause Rating Scale (MRS), which consists of 11 common symptoms, was divided into three dimensions: psychological (4 symptoms); somato-vegetative (4 symptoms); and urogenital (3 symptoms). It was used to assess the severity of symptoms. Each of the symptoms is scored according to the severity of the symptoms as perceived by the subjects themselves, ranging from 0 (no symptoms) to 4 (extremely severe). A total MRS score ranging from 0 to 44, with higher scores indicating more severe symptoms [48]. The psychometrics of MRS have been demonstrated [48], and the reliability and validity of the Traditional Chinese version of MRS have been verified [49].

Medication beliefs: The Beliefs about Medicines Questionnaire (BMQ) was used to measure medication beliefs. There are two sub-scales in the BMQ, the Specific scale and the General scale [50]. For the objectives of the MOCHA-T study, only 7 items that have high factor loading scores (> 0.7) in the General scale were used to capture the individual’s overall perception of medication use. The questionnaire used a 5-point Likert scale ranging from 1 (strongly disagree) to 5 (strongly agree) with higher scores indicating greater agreement with the beliefs corresponding to the subscale in question [50, 51]. The reliability and validity of the BMQ-General have been in evidence [50].

Mental health: This sub-questionnaire was developed to assess the level of loneliness, depressive symptoms, and life satisfaction of individuals. A reliable and valid 8-item short form of the UCLA Loneliness Scale (ULS-8) was used to measure loneliness [52]. Each item was scored using a 4-point Likert scale, ranging from 1 (never) to 4 (always), with a total score range of 8 to 32 points. Higher scores indicated a greater level of loneliness. The Chinese version of ULS-8 also had good internal reliability (0.84) and validity [53]. The level of depressive symptoms was measured by the Chinese version 10-item short-form, which was developed based on the Center for Epidemiological Studies Depression Scale (CES-D) [54]. This version possessed satisfactory psychometric properties [55]. In the scale, each item was scored on a 4-point Likert scale ranging from 0 (never) to 3 (often). The total score ranged from 0 to 30 points with higher scores indicating greater depressive symptoms. In terms of life satisfaction, the Satisfaction with Life Scale (SWLS) was used to measure personal life satisfaction [56]. This scale had five items, each ranging from 1 to 7. The total score ranges from 5 to 35, with higher scores indicating greater life satisfaction. SWLS had good reliability and validity [57, 58].

Sleep log: Every participant kept a sleep diary for 7 days to record their wake-up time, bedtime, times of wake after sleep onset, subjective sleep quality, stimulants affecting sleep (such as alcohol and caffeine) intake after 3 p.m., and sedative medications.

Daily activities: Participants recorded their daily activities and the time of activity. There were 12 categories of activity, including work, sedentary activities, meetings with relatives and friends, gardening and farming, taking a walk, shopping, housework, going for a walk in the countryside, exercising outdoors, exercising indoors, group activities, and others.

Daily mood: The daily mood was reported in the diary.

2. Objective measurements.

Anthropometry: Wight and waist circumference were measured by trained interviewers during the interview.

Sleep parameters: Every participant wore a wrist actigraphy recorder, which was developed by Kuo et al., on their non-dominant wrist for consecutive 7 days to measure their objective sleep patterns [59]. A three-axis accelerometer is used in the actigraphy recorder to record the wrist movements, which can be as personal sleep-wake detection. A total wear time of at least 7200 min was defined as valid data [60]. Raw data were processed using a window-based sleep-wake staging algorithm to generate four sleep parameters: sleep onset time (SOT), wake after sleep onset (WASO), total sleep time (TST), total wake time (TWT), and sleep efficiency (SE). The robustness and reliability of this sleep measurement have been demonstrated [59].

Rest-activity rhythms: The actigraph counts could be generated from the aforementioned acceleration raw data via a highly valid aggregation method developed by Brønd et al. [61]. Through analyzing the actigraph counts by the cosinor model and nonparametric method, the following rest-activity rhythm parameters could be generated [62, 63]. (1) Midline estimating statistic of rhythm (MESOR) was a rhythm-adjusted mean of activity in a whole day, (2) amplitude represented the distance from the MESOR to the peak, (3) acrophase was the timing of peak activity, (4) interdaily stability (IS) indicated the stability of rest-activity rhythm across the days, (5) intradaily variability (IV) indicated the fragmentation of rest-activity rhythm within the day, (6) the least active 5-h period (L5) was the average activity values during the least active five-hour period, (7) L5 midpoint was the time middle point of the least active five-hour period, (8) most active 10-h period (M10) was the average activity values during the most active ten-hour period, (9) M10 midpoint was the time middle point during the most active ten-hour period, (10) relative amplitude (RA) indicated the difference between the resting level and the activity level, and (11) chronotype represented the diurnal preferences, which was converted from acrophase [64].

Physical activities: Physical activities were evaluated by calculating and dividing the actigraph counts within a day. The valid data was defined as the total wear time was more than 80% (1152 min) during a 24-hour period. According to the definition of activity level criteria [65, 66], the physical activity levels in every person-day based on actigraph counts were categorized into moderate to vigorous physical activity (MVPA), light physical activity (LPA), and sedentary behavior (SED) time. Furthermore, the mean level of physical activity in the morning (5:00–12:00), in the afternoon (12:00–18:00), in the evening (18:00–24:00), and in the day (00:00–24:00) were also established.

GPS: Data from the GPS sensor on each person’s smartphone was used to collect location data. Raw GPS data were recorded by the Hiking Biji application on participants’ smartphones. Researchers installed the Hiking Biji application at the interview, continuously ran it in the background, and removed it when the case was closed. The GPS sample rate was 6 s per time; while the actigraphy record rate was 10 s per time. To combine two objective measurements, the researcher down sampled the sample rate to 1 min per time [67]. The GPS variables were created from Quantum Geographic Information System (QGIS) and Arc Geographic Information System (ArcGIS). Researchers implemented the Density-Based Spatial Clustering of Applications with Noise (DBSCAN) method and matched the points of interest (POI) database in OpenStreetMap [68]. Moreover, researchers also adopted the standard deviational ellipse area, daily path area, and the average distance from home to present the activity space [16, 69, 70]. The GPS validity criteria were set at more than 360 min of data recorded in a day as a valid GPS person-day. More than 2 valid GPS person-days was considered a successful case.

### Quality control

The following procedures for ensuring data quality were implemented in the MOCHA-T study. First, standardized study procedures were established to ensure the accuracy and consistency of the data collection. Second, a pilot study was performed to identify the standardized study procedures and to correct the potential problems during the data collection. Third, every interviewer needed to be trained, including standardized study procedures, study instruction, interview skills, and data collection. Fourth, using the LINE application was a connection between interviewers and participants for reporting and dealing with problems immediately. Fifth, data entering, data processing, and data management were executed by trained researchers to ensure the accuracy of all data. Lastly, interviewers reported and discussed the progress and problems with the supervisor during the study.

## Results

Table 2 shows the completion rate and summary of the main reasons for missing data for diary, actigraphy, and GPS data. The completion rates of diary, actigraphy, and GPS data were 94.6%, 95.0%, and 25.2%, respectively. The main reasons for missing diary data were forgetfulness and the perceived inconvenience of keeping a diary. For the actigraphy data, the main reasons for the lack of actigraphy data were a total wear time of fewer than 7200 min and inaccessible data due to recorder malfunctions. For the GPS application, the low response rate to having GPS data collected was the main reason for missing data. In addition, turning off the smartphone or GPS tracker during the study was another common reason.

**Table 2:** Data completion rate and reasons for missing data for diary, actigraphy, and GPS

Data	Completion rate	Reasons for missing data
Diary	94.6%	Forgetting to fill in the diary, feeling that keeping daily records is a hassle
Actigraphy	95.0%	Wearing the actigraphy recorder for less than 7200 min, raw data is inaccessible owing to recorder malfunction
GPS	25.2%	Low response rate to participate in GPS data collection, turning off the smartphone or GPS tracker during the study concerns

The sociodemographic characteristics of participants with and without a complete diary, actigraphy, and GPS data are shown in Table 3. No significant differences in most sociodemographic characteristics were found between participants with complete diaries, actigraphy, and GPS data and those without. However, the distribution of their age groups between those with and without actigraphy data was different (*p* = 0.034). For participants with actigraphy data, the largest proportion was in the 50–59 age group (50.0%), followed by the 60–69 (30.9%), while for those without actigraphy data, the largest proportion was in the 50–59 age group (66.7%), followed by the ≥ 70 age group (25.0%). More than half of the participants with actigraphy data had a college degree or higher, while about two in three of the participants without actigraphy data had a high school degree (*p* = 0.029).

**Table 3:** Sociodemographic characteristics with and without complete data on diary, actigraphy, and GPS

Variables	Diary				Actigraphy				GPS		
	w/ complete data n = 229 (94.6%)	w/o complete data n = 13 (5.4%)	t/ χ^2^		w/ complete data n = 230 (95.0%)	w/o complete data n = 12 (5.0%)	t/ χ^2^		w/ complete data n = 61 (25.2%)	w/o complete data n = 181 (74.8%)	t/ χ^2^
Sociodemographics											
Gender, n (%)			0.528^a^				0.517^a^				0.209
Men	64 (27.9)	5 (38.5)			67 (29.1)	2 (16.7)			16 (26.2)	53 (29.3)	
Women	165 (72.1)	8 (61.5)			163 (70.9)	10 (83.3)			45 (73.8)	128 (70.7)	
Age, years, *M* ± SD	58.06 ± 8.27	58.38 ± 8.75	-0.139		57.98 ± 8.08	59.83 ± 11.77	-0.538		56.69 ± 6.61	58.54 ± 8.74	1.516
Age groups, n (%)			1.000^a^				**0.034** ^**a**^				0.409^a^
45–49 (only women)	28 (12.2)	1 (7.7)			**29 (12.6)**	**0 (0.0)**			8 (13.1)	21 (11.6)	
50–59	116 (50.7)	7 (53.8)			**115 (50.0)**	**8 (66.7)**			35 (57.4)	88 (48.6)	
60–69	68 (29.7)	4 (30.8)			**71 (30.9)**	**1 (8.3)**			16 (26.2)	56 (30.9)	
≧ 70	17 (7.4)	1(7.7)			**15 (6.5)**	**3 (25.0)**			2 (3.3)	16 (8.8)	
Educational level, n (%)			0.668^a^				**0.029** ^**a**^				1.335
Junior school or less	25 (10.9)	1 (7.7)			**24 (10.4)**	**2 (16.7)**			6 (9.8)	20 (11.1)	
High school	81 (35.4)	3 (23.1)			**76 (33.0)**	**8 (66.7)**			18 (29.5)	66 (36.7)	
College or above	122 (53.3)	9 (69.2)			**129 (56.1)**	**2 (16.7)**			37 (60.7)	94 (52.2)	
Marital status, n (%)			0.684^a^				0.364^a^				0.292^a^
Married or cohabiting	174 (76.0)	11 (84.6)			117 (77.0)	8 (66.7)			52 (85.2)	133 (73.5)	
Never married	11 (4.8)	1 (7.7)			11 (4.8)	1 (8.3)			1 (1.6)	11 (6.1)	
Divorced	21 (9.2)	0 (0.0)			19 (8.3)	2 (16.7)			3 (4.9)	18 (9.9)	
Widowed	23 (10.0)	1 (7.7)			23 (10.0)	1 (8.3)			5 (8.2)	19 (10.5)	
Living arrangements, n (%)			1.000^a^				0.569^a^				1.000^a^
Living with others	213 (93.0)	13 (100.0)			215 (93.5)	11 (91.7)			57 (93.4)	169 (93.4)	
Living alone	16 (7.0)	0 (0.0)			15 (6.5)	1 (8.3)			4 (6.6)	12 (6.6)	
Employment status, n (%)			0.702^a^				0.059^a^				.210^a^
Employed	136 (59.4)	9 (69.2)			140 (60.9)	5 (41.7)			41 (67.2)	104 (57.5)	
Retired	55 (24.0)	2 (15.4)			55 (23.9)	2 (16.7)			14 (23.0)	43 (23.8)	
Housemaker	29 (12.7)	1 (7.7)			27 (11.7)	3 (25.0)			6 (9.8)	24 (13.3)	
No job	9 (3.9)	1 (7.7)			8 (3.5)	2 (16.7)			0 (0.0)	10 (5.5)	

^a:^*p*-value calculated with Fisher’s Exact Test; Bold numbers indicate significance at p < 0.05 level

## Discussion

The MOCHA-T study aims to investigate the health, behaviors, and lifestyles of middle-aged and older Chinese adults in Taiwan using multidimensional instruments. To our best knowledge, it is the first study to develop a multi-method investigation combining questionnaires, diaries, actigraphy, and GPS to comprehensively collect both subjective and objective health data from this population. The survey covered multiple dimensions, including nutrition, exercise, social participation, illness, medication, mental health, sleep, and activity. This variety of elements allows researchers to identify the relationship between health and behaviors from different perspectives.

The study population were a unique feature of the MOCHA-T study. By enrolling people aged 50 and above, the MOCHA-T study was able to show the profile of middle-aged and older adults in different age groups. With participants spanning several generations, including the Silent Generation, Baby Boomers, and Generation X, this study has also been able to characterize generational differences in health and lifestyle patterns. This study also extended the recruitment of women aged 45–49 and examined gynecological health variables. This helped to add women’s health to the field of aging research and provided insights into how both the menopause process and aging contribute to health outcomes in later life.

The completion rate of diary and actigraphy data was both over 90%. With regard to GPS data, however, the completion rate was only 25.2%. The explanations for the low completion rate can be divided into two components: high missing data and high invalid data. High levels of missing data may be related to low uptake of GPS application use and incompatibility between mobile devices and applications. Some participants only agreed to complete the questionnaire and wear the actigraphy recorder but were concerned that the GPS data collection would reveal their personal information, resulting in a lower response rate. Furthermore, participants who declined GPS data collection may perceive themselves as less tech-savvy and tend to maintain their regular usage patterns. This could lead to resistance if the interviewers asked them to download and use an unfamiliar application during enrolment. Some smartphones were unable to download the GPS application used in the study due to an outdated system version, which also increased the missing data. Additionally, smartphone usage behaviors are related to the barriers to GPS data collection which increases the amount of invalid data. Turning off the smartphone is a common behavior in the population and is positively correlated with age [71]. This behavior resulted in GPS data not being recorded. Despite the low completion rate of GPS data in this study, the findings shed light on the difficulties of collecting GPS data using smartphones in middle-aged and older adults.

There were no significant differences in most sociodemographic characteristics between those with and without diary, actigraphy, and GPS data, except for the age group and educational level for actigraphy. The proportion of participants in the 70 + age group who did not complete actigraphy data was higher than the proportion of people who completed actigraphy data. This may be because the actigraphy recorder used in this study had to be taken off during activities involving water exposure, older adults might forget to put it back on due to age-related cognitive decline. This could lead to prolonged periods of not wearing the device, resulting in insufficient data and missing data cases. In addition, participants with complete actigraphy data in our study had a higher level of education compared to those without complete actigraphy data. These findings different from the previous study in older adults found that adherence to using wearable activity trackers was not significantly related to educational level [72]. In our study sample, 45% of participants had a high school education or less, and many had no previous experience with wearables, which may therefore highlight the importance of higher education in completing the data collection on the novel wearable recorders as instructed. It is possible to gradually reduce the impact of the educational gap on data collection as the education of middle-aged and older adults has generally increased. However, since the educational gap is still an important factor in the acceptance of wearable technology [73], recruiting people with low levels of education in studies using actigraphy is an important issue for the future.

Some limitations existed in this study. Convenience sampling in each community could potentially introduce selection bias, as participants who were more concerned about their health status or more interested in new study tools were more likely to participate in this study. Furthermore, some participants were not available for face-to-face interviews due to geographical factors. The researcher overcame this by using study kits. However, although a voice or video call instruction of the study may reduce the difference between a face-to-face interview and receiving a kit, there may still be unavoidable biases in the results. Lastly, high completion rates of diary and actigraphy were investigated in the MOCHA-T study. It should be noted that these high completion rates could be attributed to the fact that participants who agreed to join the study had already consented to the collection of diary and actigraphy data.

Despite the aforementioned challenges and limitations, the MOCHA-T study exhibited several strengths. The strength of this study was that it provided objective data on sleep, physical activity, rest-activity rhythm, and GPS for each person at different times of the day. The objective data collected in this study compensated for the lack of data from subjective questionnaires and extended the application of this dataset. Furthermore, adding the type of activities recorded by the participants in their diaries can also help to identify the relationship between different types of activities, location preferences, and levels of activity. Not only providing subjective and objective data among older adults of the new generation, the MOCHA-T study also provided the sociodemographic characteristics of participants who completed the different types of data or not. These results can inform other researchers interested in this dataset how to adjust the data, as well as showing the potential individual bias of different types of data. In short, with the temporal and spatial data, the MOCHA-T study has the potential to reveal the complex interactions between a person and the environment in later adulthood.

The dataset from the MOCHA-T study has the potential for a variety of applications. First, with the multidimensional data collected in this dataset, researchers can explore the connections among the individual’s health, lifestyle and environment of middle-aged and older Chinese adults. For instance, combining actigraphy to objectively measure circadian rhythm, physical activity, and sleep with self-reported data can help investigate plausible associations between sociodemographic characteristics [74–76], physical and mental health [20, 77, 78], and behaviors [75]. In addition, the inclusion of temporal and spatial information from GPS can identify the range of time and space in the daily activities of older adults, facilitating further exploration of the interaction between older adult behavior and the environment [79]. This allows researchers to examine associations such as mobility with health and well-being [80], and the relationship between space, environment, and behaviors [81, 82]. Furthermore, by categorizing middle-aged and older adults according to their behaviors, different lifestyle groups can be identified and their associated health outcomes can be studied. Individuals can also be categorized into groups with different backgrounds according to their socio-demographic characteristics and health conditions. This sheds light on diverse lifestyle patterns and allows for the identification of potentially appropriate health promotion strategies in different groups.

There is also abundant room for extension of this study along the line. First, supplement samples in specific scenarios, such as Generation X, Baby Boomers, those living in rural areas, and those in caring institutions to compare the differences in lifestyle patterns. Second, because health is influenced by individuals, communities, human-made environment, culture, biosphere, and their interactions [83], a cross-national dataset is necessary to be established. The collections of middle-aged and older Chinese adults in different countries and people of different ethnicities allow the role of country context, culture, and ethnicity as modifiers of health and lifestyle patterns to be identified. Finally, the MOCHA-T study will be refined and made more valuable in the future through collaboration with other studies. For example, comparisons with other studies using actigraphy, such as the National Social Life, Health, and Aging Project (NSHAP) [84] and the UK biobank study [85, 86], to determine objective sleep and physical activity patterns among middle-aged and older adults in different countries. Furthermore, leverage other lifestyle and population health studies, such as the CARTaGENE study [87] and the Canadian Partnership for Tomorrow Project [88], as references to continually expand the breadth and depth of data, and conduct comparisons. By adding physical measurements, biological samples, family and neighborhood variables, as well as linking with government health and environmental databases, the MOCHA-T study will be able to discern health determinants from both micro and macro perspectives.

## Conclusions

In conclusion, the MOCHA-T study is an innovative and promising study conducted in Taiwan that used multiple instruments to obtain multidimensional data. Combined with these multiple data, this study can provide a dataset that allows researchers to analyze the association and interaction between physical and psycho-social health, behaviors, and the environment. Findings from the MOCHA-T study can advance knowledge about healthy aging targeted to the Chinese older population and provide health promotion suggestions based on personal health and lifestyle patterns.

## Data Availability

The datasets used and analysed during the current study are available from the corresponding author on reasonable request.

## Abbreviations

ArcGIS: Arc Geographic Information System
BMQ: Beliefs about Medicines Questionnaire
CES-D: Center for Epidemiological Studies Depression Scale
CURHA: Contrasted Urban settings for Healthy Aging
DASH: Dietary approaches to stop hypertension
DBSCAN: Density-Based Spatial Clustering of Applications with Noise
IS: Interdaily stability
IV: Intradaily variability
L5: Least active 5-h period
LPA: Light physical activity
GPS: Global Positioning System
M10: Most active 10-h period
MESOR: Midline estimating statistic of rhythm
MOCHA-T: Middle-aged and older Chinese adults’ health using actigraphy in Taiwan
MRS: Menopause Rating Scale
MVPA: moderate to vigorous physical activity
NSHAP: National Social Life, Health, and Aging Project
POI: Points of interest
QGIS: Quantum Geographic Information System
QR code: Quick response code
RA: Relative amplitude
SE: Sleep efficiency
SED: Sedentary behavior
SOT: Sleep onset time
SWLS: Satisfaction with Life Scale
TST: Total sleep time
TWT: Total wake time
ULS-8: 8-item short-form UCLA Loneliness Scale
WASO: Wake after sleep onset
